# Decoding the key compounds and mechanism of Shashen Maidong decoction in the treatment of lung cancer

**DOI:** 10.1186/s12906-023-03985-y

**Published:** 2023-05-15

**Authors:** Jieqi Cai, Yupeng Chen, Kexin Wang, Yi Li, Jie Wu, Hailang Yu, Qingping Li, Qi Wu, Wei Meng, Handuo Wang, Aiping Lu, Mianbo Huang, Genxia Wei, Daogang Guan

**Affiliations:** 1 Department of Biochemistry and Molecular Biology, School of Basic Medical Sciences, Southern Medical University, Guangzhou, Guangdong Province China; 2 Guangdong Provincial Key Laboratory of Single Cell Technology and Application, Guangzhou, Guangdong Province China; 3 Neurosurgery Center, Guangdong Provincial Key Laboratory on Brain Function Repair and Regeneration, Department of Cerebrovascular Surgery, Engineering Technology Research Center of Education Ministry of China on Diagnosis and Treatment of Cerebrovascular Disease, Zhujiang Hospital, Southern Medical University, Guangzhou, Guangdong 510280 China; 4 Division of Hepatobiliopancreatic Surgery, Department of General Surgery, Nanfang Hospital, Southern Medical University, Guangzhou, Guangdong China; 5 Department of Burns, Nanfang Hospital, Southern Medical University, Guangzhou, China; 6 Institute of Integrated Bioinformedicine and Translational Science, Hong Kong Baptist University, Hong Kong, China; 7 Department of Histology and Embryology, School of Basic Medical Sciences, Southern Medical University, Guangzhou, Guangdong Province China; 8 Huiqiao Medical Center, Nanfang Hospital, Southern Medical University, Guangzhou, China

**Keywords:** Shashen Maidong decoction, Lung cancer, Network analysis, Key functional networks, Contribution decision rate

## Abstract

**Background:**

Lung cancer is a malignant tumour with the fastest increase in morbidity and mortality around the world. The clinical treatments available have significant side effects, thus it is desirable to identify alternative modalities to treat lung cancer. Shashen Maidong decoction (SMD) is a commonly used traditional Chinese medicine (TCM) formula for treating lung cancer in the clinic. While the key functional components (KFC) and the underlying mechanisms of SMD treating lung cancer are still unclear.

**Methods:**

We propose a new integrated pharmacology model, which combines a novel node-importance calculation method and the contribution decision rate (CDR) model, to identify the KFC of SMD and to deduce their mechanisms in the treatment of lung cancer.

**Results:**

The enriched effective Gene Ontology (GO) terms selected from our proposed node importance detection method could cover 97.66% of enriched GO terms of reference targets. After calculating CDR of active components in key functional network, the first 82 components covered 90.25% of the network information, which were defined as KFC. 82 KFC were subjected to functional analysis and experimental validation. 5–40 μM protocatechuic acid, 100–400 μM paeonol or caffeic acid exerted significant inhibitory activity on the proliferation of A549 cells. The results show that KFC play an important therapeutic role in the treatment of lung cancer by targeting Ras, AKT, IKK, Raf1, MEK, and NF-κB in the PI3K-Akt, MAPK, SCLC, and NSCLC signaling pathways active in lung cancer.

**Conclusions:**

This study provides a methodological reference for the optimization and secondary development of TCM formulas. The strategy proposed in this study can be used to identify key compounds in the complex network and provides an operable test range for subsequent experimental verification, which greatly reduces the experimental workload.

**Supplementary Information:**

The online version contains supplementary material available at 10.1186/s12906-023-03985-y.

## Introduction

Lung cancer is the most common primary malignancy and ranks first in the incidence and mortality of malignant tumors in the world. The incidence of lung cancer has reached 11.6% of total cancer cases, and mortality is 18.4% of total cancer deaths [1]. At present, lung cancer treatments mainly include surgery, radiation, and chemotherapy. These treatments can prevent the malignant development of tumor cells and improve survival time. However, most treatments present obvious side effects in clinical use, and can cause serious or general damage to specific or all tissues, including functional damage to the digestive system and urinary system [2]. In addition to the above treatments, some targeted drugs are often used in the clinical treatment of lung cancer, including gefitinib [3], bevacizumab [4], and osimertinib [5], but with these drugs it is difficult to control toxicity and side effects. Thus, it is desirable to improve the treatment for lung cancer.

Many clinical studies have shown that anti-lung cancer treatment combined with Traditional Chinese Medicine (TCM) can reduce the side effects of radiotherapy and chemotherapy, improve therapeutic effects, and reduce complications. Meanwhile, TCM can prolong the life of lung cancer patients and improve their survival quality of life [6]. Currently, some studies have shown that TCM formulas could effectively treat lung cancer. For example, a well-known Yangyinwenyang formula was used to induce lung cancer cell apoptosis in vitro [7]. Jinfukang’s formula could inhibit lung cancer cell proliferation by promoting cell apoptosis [8]. Kangliuzengxiao decoction is successful for the prevention the development of tumors and improving major clinical symptoms [9]. Shashen Maidong Decoction (SMD) can alleviate symptoms of patients and can reduce the inflammatory response [10]. Among these formulas, SMD is widely used in the clinical treatment of lung cancer. As reports, based on the cancer toxicity theory, the application of SMD in treating lung cancer cachexia has definite therapeutic effects and important clinical values [10]. The carcinoembryonic antigen (CEA) and carbohydrate antigen 153 (CA 153) level was significantly reduced in serum of two patient groups [11]. In addition, SMD is proved to have obvious inhibition on the growth of A549 cells. In the result of MTT assay, the growth of A549 cells was inhibited obviously in the SMD-containing serum [12, 13]. A549 cells cultured in the SMD-containing serum showed higher E-cadherin protein expression, lower Snail protein expression (*P* < 0.05) [12], higher Smad7 protein expression and lower TGF-β1 protein expression [13].

SMD is composed of 7 herbs, *Glehnia littoralis* (A.Gray) F.Schmidt ex Miq. (*G. littoralis*, Beishashen, 9 g), *Ophiopogon japonicus* (Thunb.) Ker Gawl. (*O. japonicus*, Maidong, 9 g), *Polygonatum odoratum* (Mill.) Druce (*P. odoratum,* Yuzhu, 6 g), *Trichosanthes kirilowii* Maxim. (*T. kirilowii*, Tianhuafen, 4.5 g), *Lablab purpureus* subsp. *purpureus* (*L. purpureus,* Baibiandou, 4.5 g), *Morus alba* L. (*M. alba,* Sangye, 4.5 g), and *Glycyrrhiza uralensis* Fisch. ex DC. (*G. uralensis*, Gancao, 3 g). *O. japonicus* and *G. littoralis* are the main drugs of SMD, and may be responsible for the therapeutic effects of SMD in lung cancer. Modern pharmacological research indicates that the extract of *O. japonicus* has an obvious inhibitory effect on the occurrence and development of lung cancer, and it can induce autophagy of A549 lung cancer cells [14]. *G. littoralis* exerts anticancer activity and induces cycle arrest of A549 cells [15]. In clinical research, SMD combined with hormones or antibiotics is a remarkable treatment for radiation pneumonia [16]. Furthermore, chemotherapy combined with modified SMD has been shown to be highly effective in the cure of mid- and late-stage non-small cell lung cancer (NSCLC) [17]. But there is still a lack reports characterizing key functional components (KFC) and the potential therapeutic mechanism of SMD on lung cancer at a system level.

Due to the “multi-components-multi-targets” of the formula, it is difficult to explore the potential associations among TCM components, target genes, and diseases using traditional experiments. Based on the theory of systems biology, integrated pharmacology can clarify the synergistic mechanism of the components-targets and of the targets-pathogenic network [18]. It is important to study the mechanisms underlying the therapeutic activity of TCM. The “multi-components-multi-targets” characteristic of TCM formulas determine that it could form complex regulation relations between components and targets in the treatment process. Among these complex relationships, some relationships have positive effects on treatment, while others have antagonistic or toxic effects. The aim of formula optimization is a process that finds components with synergistic effects and removes components with antagonistic or toxic effects [19]. Optimization of the formula would be beneficial for the secondary development of TCM.

**Fig. 1:**
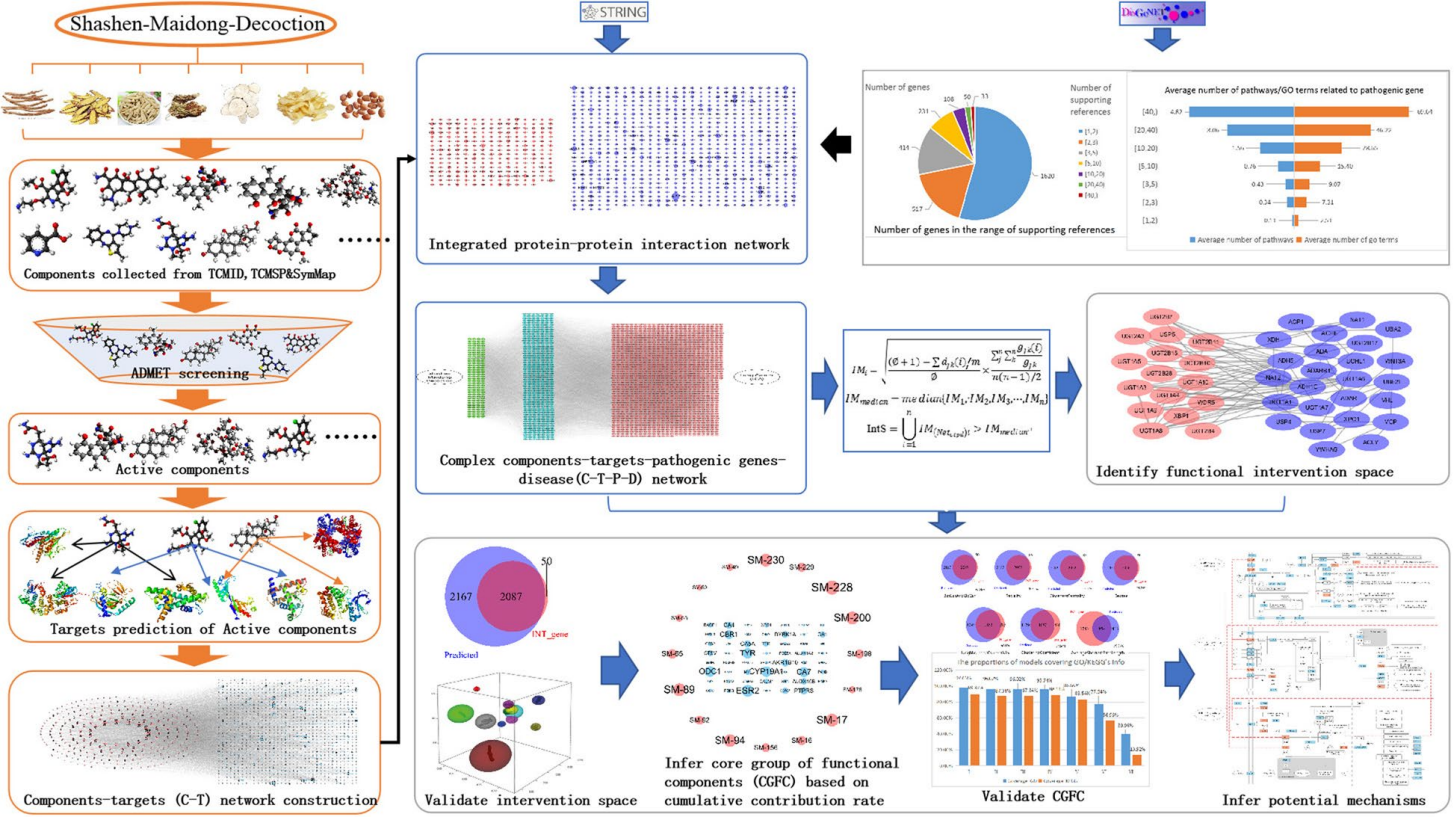
The work scheme of our proposed integrated pharmacology approach

In this study (Fig. 1), the chemicals of SMD were collected from published databases, then the active components were screened from all components based on Lipinski’s rule, oral bioavailability (OB) value, and gastrointestinal (GI) absorption. The targets of active components were predicted using on-line tools, the active components and their targets were used to construct the components-targets (CT) network. Meanwhile, pathogenic genes associated with lung cancer were collected from DisGeNET [20] to construct a network of weighted pathogenic genes. The CT network and the weighted pathogenic gene network were then integrated to construct the CTP network. Finally, a node importance calculation method was designed to select the key functional network and effective proteins. The contribution decision rate (CDR) model was developed to capture KFC. Finally, KFC and their targets were used to infer the potential mechanism of SMD in the treatment of lung cancer.

## Materials and methods

### Identification of the components of Shashen Maidong decoction and chemical analysis

The component structures of SMD in MOL2 format were downloaded from the Traditional Chinese Medicine Systems Pharmacology Database and Analysis Platform (TCMSP) [21], the Encyclopedia of Traditional Chinese Medicine (ETCM) [22], and Symptom Mapping (SymMap) [23]. The components in different formats were converted into a unified SMILES format by Open Babel 2.4.1 [24]. We searched for experimentally validated high concentrations and biological activities of chemical components of SMD from the literature to obtain a comprehensive list of active components.

### Selection of potential active components based on published ADMET models

In general, effective drugs have good absorption, distribution, metabolism, excretion, and toxicity (ADMET) properties. Therefore, in the process of screening possible active components of SMD, we considered some ADMET properties, including Lipinski’s rule [25], GI absorption value [26], and OB [21].

The detailed criteria of Lipinski’s Rule are as follows: there are no more than 5 H-bond donors, less than 10 H-bond acceptors, the molecular weight (MW) is not greater than 500, the calculated Log P is not greater than 5 and the number of rotatable bonds in compounds is less than 10. Ingredients that meet these standards have better drug-like properties.

OB (%F) represents the percentage of oral dose of the chemical components of botanical drugs that are released into the systemic circulation. The chemical components with OB ≥ 30% are well absorbed in human body, so these chemical components were selected for further study.

GI absorption is a pharmacokinetic property, and it is vital to estimate the GI absorption value at each stage of the drug discovery process. GI absorption values of all components in SMD were obtained from SwissADME and chemical components with high GI values were retained for further research.

### Predicting the molecular targets of the active components

To obtain the molecular targets of the active components in SMD, we used SwissTargetPrediction [27], HitPick [28], and the Similarity Ensemble Approach (SEA) [29] to predict the potential molecular targets.

### Constructing the weighted pathogenic genes network of lung cancer

In the DisGeNET database [20], we identified a total of 27 lung cancer-related international classification of diseases (ICD, version 10) codes by querying “lung cancer” as the key word. We then used these classification IDs to query related genes; a total of 2973 pathogenic genes were obtained.

### Definition of protein–protein interaction data

We submitted 2973 pathogenic genes and 1221 predicted targets of active components to the STRING [30] database and chose “Homo sapiens” as the species parameter to obtain the protein–protein interaction (PPI) network.

### Construction of component-target network

All SMD chemicals collected in the database were screened with ADMET to obtain active components, and the targets of these active components were predicted by Hitpick, SwissTargetPrediction, and SEA. Active components and their predicted targets were used to construct components-targets (CT) network using Cytoscape [31].

### Screening of key functional networks

We constructed a four-layer component-target-gene-disease network by combining CT, PPI network, and pathogenic gene network, and we determined and verified the key component-target-pathogenic gene associations in the network with our newly designed node-importance calculation method. The detailed algorithm was described in the Supporting information.

### Prediction of key functional components

The Contribution Decision Rate (CDR) model, which was employed and modified based on knapsack algorithm, was used to determine the KFC in the network: Assuming there is a function that can solve the total value with two dependent variables n and C, the detailed algorithm was described in the Supporting information.

### GO and KEGG analysis

R package clusterProfiler [32] was used to perform the Kyoto Encyclopedia of Genes and Genomes (KEGG) [33–35] and Gene Ontology (GO) enrichment analysis, and *P*-value < 0.05 was set as significant level. After that, we used ggplot2 [36] to create the graph of enrichment results. Then, some specific and enriched pathways were merged and visualized to speculate on the potential mechanism of SMD on lung cancer.

### Cell culture and treatment

Human lung cancer A549 cells were obtained from GuangZhou Jennio Biotech Co., Ltd. Fetal bovine serum (FBS) and Dulbecco’s modified Eagle’s medium (DMEM) were purchased from Gibco (Grand Island, USA). A549 cells were cultured in DMEM, containing 10% FBS, 100 units/mL of penicillin, and 100 μg/mL of streptomycin at 37℃ under 5% CO2. Protocatechuic acid (≥ 99.5% purity by HPLC), paeonol (≥ 99% purity by HPLC), and caffeic acid (≥ 98% purity by HPLC), which purchased form Jiangsu Yongjian Pharmaceutical Technology Co.,Ltd, were dissolved in DMSO. When the cells reached 80% confluence, they were treated with various concentrations of protocatechuic acid (1, 5, 10, 20, 40 μM), paeonol (25, 50, 100, 200, 400 μM) or caffeic acid (25, 50, 100, 200, 400 μM) for 24 h, respectively. The control group was not treated with drug components.

The concentration of our experimental components was determined referring to public reports. As reported, 80 or 100 μg/mL Paeonol caused a significant effect on cell viability of A549 cells compared to 0 μg/mL Paeonol [37, 38]. Caffeic acid with a concentration higher than 100 μM has significant cytotoxic effect on A549 cells [39]. Protocatechuic acid at 2–8 micromol/L significantly inhibited A549 cell adhesion (*P* < 0.05) [40].

### Cell viability assay

A549 cells (1 × 105 cells/well) were planted in 96-well plates. After a 24-h culture, the cells were treated with different concentrations of protocatechuic acid (1, 5, 10, 20, 40 μM), paeonol (25, 50, 100, 200, 400 μM), or caffeic acid (25, 50, 100, 200, 400 μM) for 24 h, respectively. Subsequently, each well was added with 10 μL of MTT solution (5 mg/ml). After 4 h, the supernatant was removed and 100 μL of DMSO was supplied into each well to dissolve the MTT formazan product. The absorbance was measured at 570 nm using the Infinite M200 PRO plate reader (Tecan, Switzerland).

## Results

### Collection of chemical components and screening of active components in SMD

**Fig. 2:**
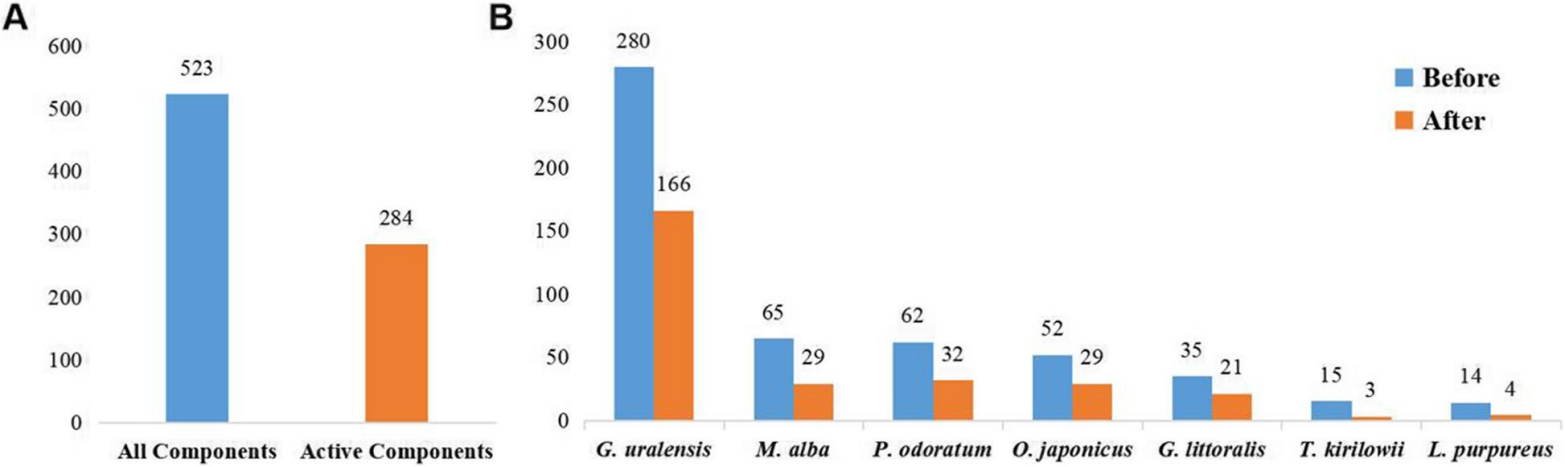
Distribution of all components and active components. **A** All components and active components of SMD. **B** All components and active components of each herb in SMD

A total of 523 types of chemical components of SMD were retrieved from TCMSP, ETCM, and SymMap databases (Additional Table 1). Among these components, some had higher concentration than expected in SMD by UPLC-MS/MS method (Table 1) [41], such as rutin, liquiritin, psoralen, xanthotoxin, bergapten, monoammonium glycyrhizinate, ophiopogonin D, methylophiopogonanone A, and methylophiopogonanone B. Previous reports confirmed that high-concentration components of SMD may play important roles in the treatment of lung cancer. By combining the experimental validated high-concentration components (Table 1) [41], and ADMET model predicted components, 284 active components were figured out for subsequent analysis (Additional Table 2, Fig. 2).

**Table 1:** The UPLC validated high-concentration components (μg/mL). ($\overline{\mathrm x}$ ± s, *n* = 3)

Compounds	Concentration (μg/mL)
Rutin	9.47 × 10^–1^ ± 1.54 × 10^–2^
Liquiritin	17.78 ± 7.65 × 10^–1^
Psoralen	1.28 × 10^–2^ ± 0.15 × 10^–3^
Xanthotoxin	3.10 × 10^–1^ ± 6.41 × 10^–2^
Bergapten	4.20 × 10^–1^ + 6.90 × 10^–2^
Monoammonium Glycyrrhizinate	24.36 ± 2.09
Ophiopogonin D	4.16 × 10^–2^ ± 7.76 × 10^–3^
Methylophiopogonanone A	2.23 × 10^–2^ ± 5.23 × 10^–3^
Methylophiopogonanone B	8.12 × 10^–2^ ± 1.59 × 10^–3^

**Fig. 3:**
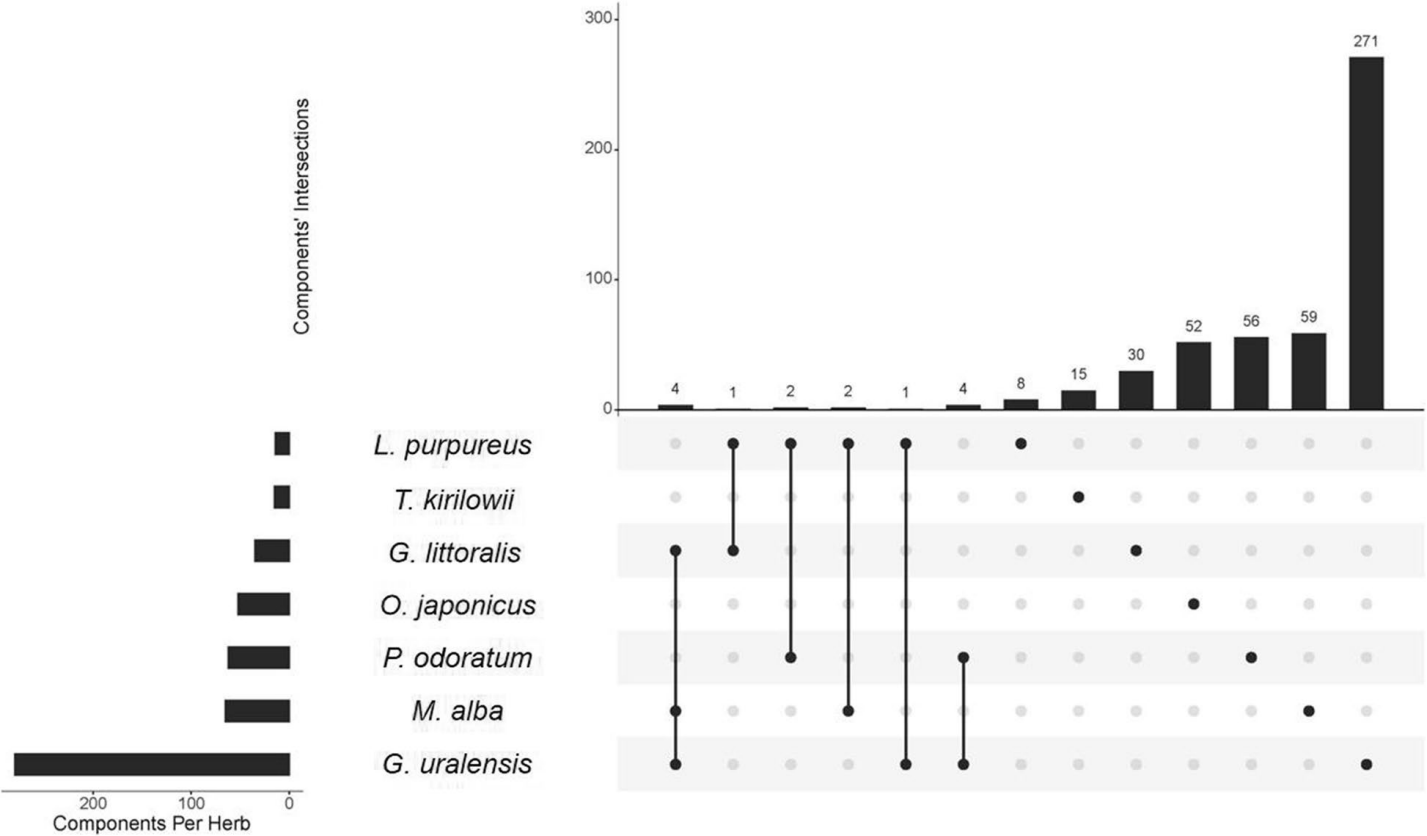
Common and specific active components in different herbs of SMD. The histogram shows the number of components common to different herbs, and the dark dots represent the original herb of the intersection

Among all active components, 14 components shared two or multiple herbs in SMD (Fig. 3), quercetin, isoquercitrin, rutin, scopoletol, glucuronic acid, 2-pentylfuran, beta-carotene, gynesine, arachic acid, 2-heptanone, linoleic acid, palmitic acid, oleic acid, and octadiene, have been shown to have clear anticancer effects. For example, quercetin has definite anticancer activity and can inhibit a variety of carcinogenic signaling pathways [42]. Rutin promotes the apoptosis of TNF-α-induced A549 human lung cancer cells [43]. Scopoletol can play an anticancer role by triggering apoptosis, blocking the cell cycle, inhibiting cell invasion, and regulating the PI3K/AKT signaling pathway [44]. Quercetin, isoquercitrin, rutin, and scopoletol are also present in *G. littoralis*, *M. alba* and *G. uralensis*, which may be of great importance in the therapeutic mechanism of SMD on lung cancer. These results indicate that shared components may play important therapeutic roles in lung cancer.

### Predicting targets of active components in SMD

**Fig. 4:**
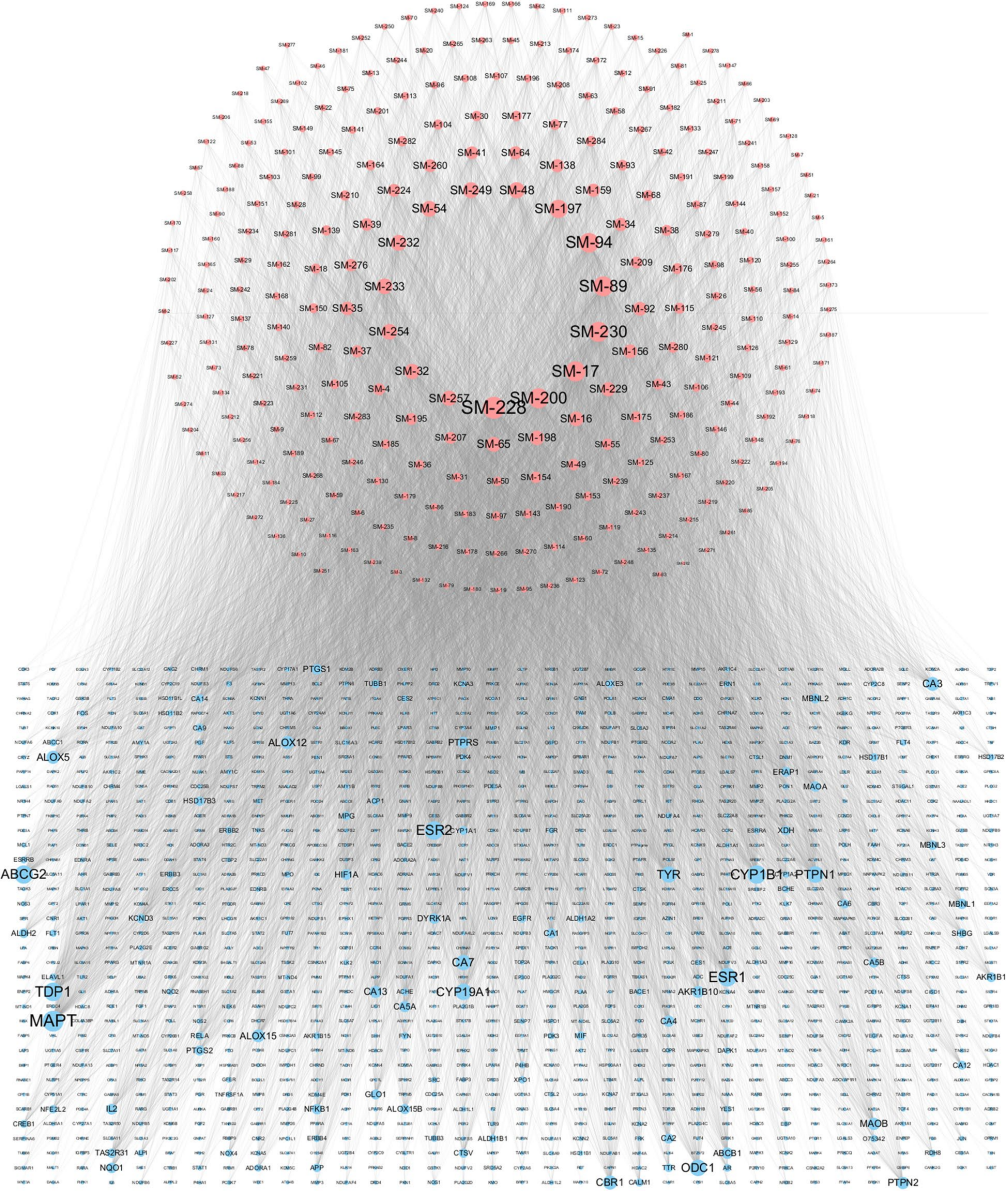
Pharmaceutical component-target interaction network. The red nodes represent pharmaceutical components, and the blue nodes refer to the targeted genes. The size of the nodes represents their degree

The 1221 targets of 284 active components were predicted by SwissTargetPrediction, HITPICK, and SEA (Additional Table 3). The active components and their targets were used to construct the CT network. This network contains 1505 nodes and 11,505 interactions (Fig. 4). We analyzed the degree of each component and each target in this network and found that the average degree of the components was 40.51 and the average degree of the targets was 9.42. Ten components with the highest degree were vanillic acid, ferulic acid, dibenzoylmethane, butyl benzoate, lauric acid, nicotinic acid, dibutyl phthalate, salicylic acid, eugenol, n-cis-feruloyltyramine. Most have been reported to be associated with cancer therapy, vanillic acid has antioxidant activity in scavenging free radicals, and thus, it has an effective preventive effect on lung cancer [45]. Lauric acid can be used as a carrier of targeted drugs due to its specific accumulation in lung cancer tissues [46]. The ten targets with the highest degree of the components were MAPT, ESR1, TDP1, ESR2, CYP1B1, ABCG2, ODC1, PTPN1, CYP19A1, and TYR. Most of these genes are reported to be related to the pathogenesis of lung cancer. MAPT has been shown to induce lung cancer cells to gain taxol resistance by activating the PI3K/Akt signaling pathway [47]. ESR1 is a central gene of lung cancer and promotes the occurrence of lung cancer by regulating the p53 signaling pathway and the cell surface receptor signaling pathway [48]. Overexpression of TDP1 is closely related to tumorigenesis, and is a crucial target for tumor treatment, the TDP1 inhibitor can significantly increase the antitumor effect of drugs [49]. These analyses demonstrate that one component can regulate multiple targets and in turn, one target is regulated by multiple components, which reflects the characteristics of the “multicomponents-multitargets” theory in treating complex diseases of TCM.

### Construction of the pathogenic genes weighted network

**Fig. 5:**
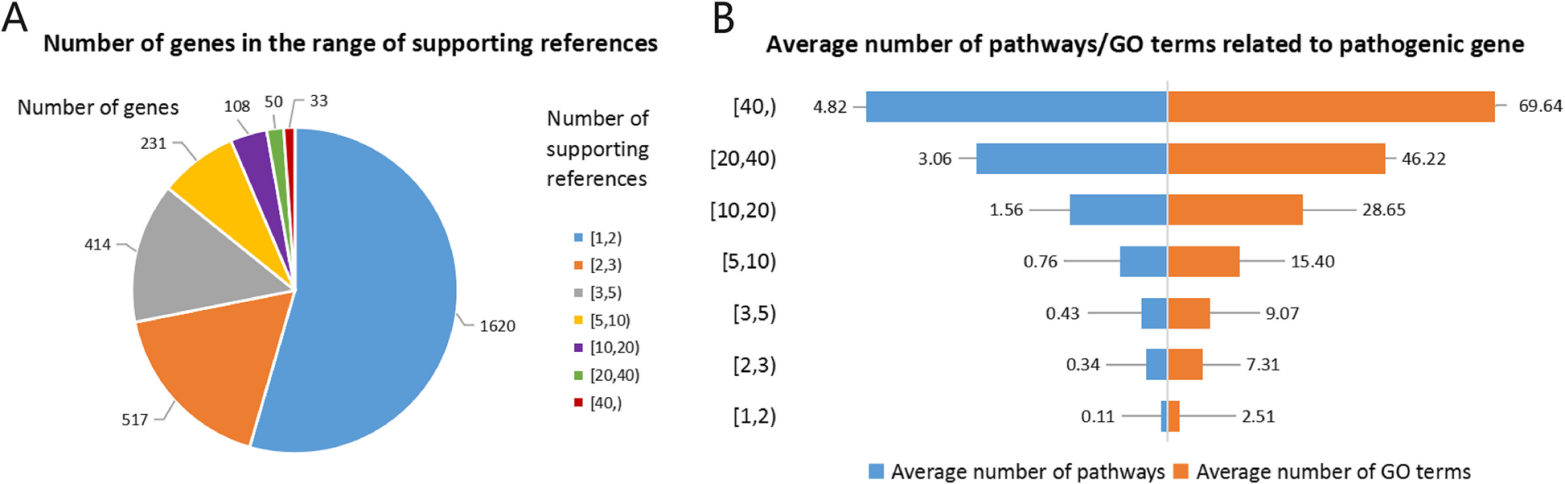
The number of supporting publications and involved pathways of pathogenic genes in lung cancer. **A** The number and distribution of supporting publications; **B** Average number of pathways and GO terms related to pathogenic gene in a distinct interval of supporting publications

The pathogenic gene data set was retrieved and downloaded from the DisGeNET database and 2973 pathogenic genes with the number of supporting publications greater than or equal to the mean value were retained (Additional Table 4). The number of supporting publications reflects the correlation of genes associated with lung cancer. A higher number of supporting publications suggests that genes have more associations with lung cancer. After counting the number of supporting publications for 2973 pathogenic genes (Fig. 5A), we found that more than half of these pathogenic genes have only one supporting reference and 33 pathogenic genes with more than 40 supporting references. The top ten genes with the highest number of supporting publications are EGFR, TP53, KRAS, ALK, GSTM1, CDKN2A, CYP1A1, ERBB2, BCL2, and MET. The epidermal growth factor receptor (EGFR) is a transmembrane glycoprotein of the ErbB family of tyrosine kinase receptors, and activated mutations of EGFR are a remarkable feature of lung cancer [50]. EGFR-activating mutations are highly sensitive to tyrosine kinase inhibitor gefitinib, so gefitinib is commonly used in clinical targeted treatment of NSCLC [51]. TP53-induced glycolytic phosphatase (TIGAR) is a key regulator of glycolysis and apoptosis, which can protect cells from oxidative stress-induced apoptosis and provide the necessary conditions for the survival of cancer cells [52]. Mutations exist widely in many types of lung cancer [53, 54]. KRAS is a potential oncogene and has been reported to have a high mutation rate, which makes cancer cells escape apoptosis-induced cell death [55]. The rearrangement of anaplastic lymphoma kinase (ALK) plays an important role in promoting the occurrence and development of lung cancer [56], thus it becomes an important clinical targeted treatment of ALK-positive NSCLC [57]. To explore whether it is reliable to measure the importance of gene function by the number of relevant supporting publications, we constructed a KEGG and GO analysis of pathogenic genes of lung cancer (Fig. 5B). The results showed that there is a positive correlation between supporting publications and the functional pathways involved by these genes, as well as GO terms. Genes with more supporting publications are associated with a highest number of pathways involved.

### Key functional network selection and validation

In formulas that treat complex diseases, some components play major therapeutic roles, some play auxiliary roles, and others play antagonistic roles. The group of components with major therapeutic roles is usually considered KFC. The KFC and their targets form key functional network, embedded within the complex CT network. How to detect the key functional network and KFC is the basis for optimizing TCM formulations. Additionally, the process of drug treatment of diseases is a continuous process of drug interventions through protein–protein interactions. Based on this information, we integrated the CT network and pathogenic gene weighted network to construct the comprehensive CTP network (Additional Fig. 1). Then we designed a new node importance calculation method to capture the key functional network. The nodes larger than the median important values of all nodes in the network were retained, and these nodes and their interactions were defined as the key functional network.

**Fig. 6:**
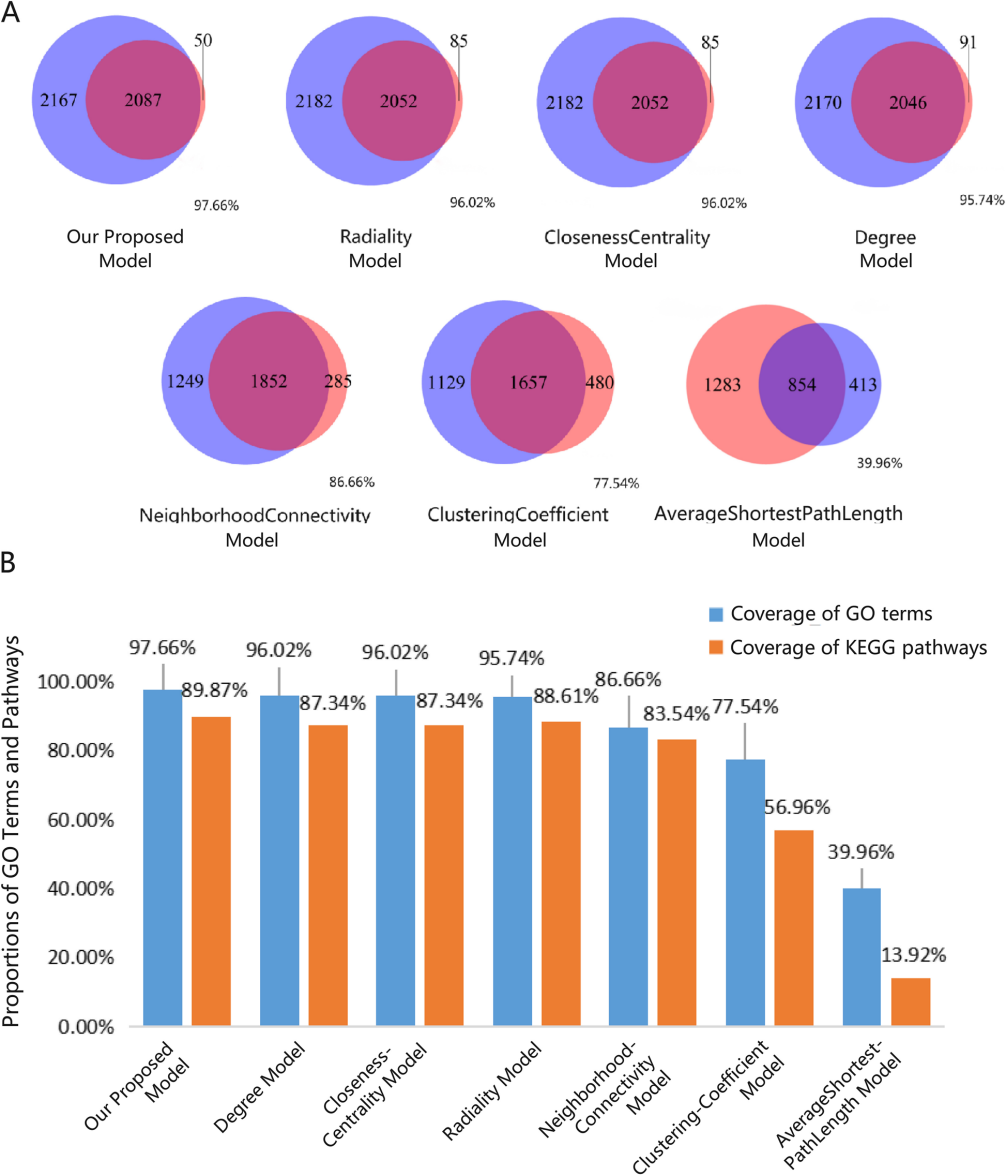
Validation of key functional networks. **A** The Venn diagrams display the number of elements of the seven models that overlap with effective GO terms. The red cycle represents the effective GO terms, and the blue cycle represents the GO terms predicted by different models. **B** Comparison of our proposed models with other traditional models on the coverage of enriched GO terms and pathways

To test the reliability of the node importance calculation method, we first performed GO enrichment analysis on targeted genes and pathogenic genes of lung cancer and considered the intersection of GO terms as the effective GO terms to serve as a reference for further comparison. Comparing our proposed node importance detection method with the other traditional methods (Fig. 6A), such as Radiality, Closeness to center, Degree, Neighborhood Connectivity Clustering coefficient and Average Shortest Path length, we found that our method covered up to 97.66% of effective GO terms, which is higher than Radiality 96.02%, Degree 95.74%, Neighborhood Connectivity 86.66%, and the Clustering Coefficient 77.54%. Figure 6B shows that our model also covered the most pathways, thus it indicated that the key functional network detection model we designed could retain the key intervention information.

### Find key functional components

**Fig. 7:**
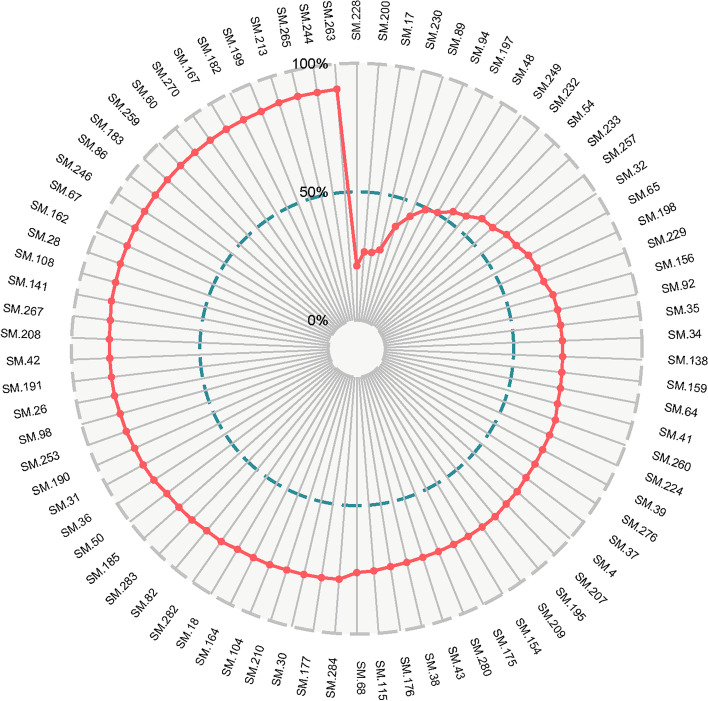
The CDR model of active components in lung cancer

After extracting the key functional network, we used the CDR model to deduce which components could retain the key functional network information to the maximum extent. Finally, we obtained 82 components that defined the KFC (Additional Table 5, Fig. 7). In the KFC, the CDR of the first 8 components reached 50% target coverage, and the 82 components reached 90% target coverage. Among these components, vanillic acid has been reported to have antioxidant activity in scavenging free radicals and had a significant and effective preventative role in B(a)P-induced lung cancer [45, 46]. Lauric acid is a good carrier of targeted drugs for lung cancer [46] and can also inhibit the expression of carcinogenic miRNA and significantly up-regulate the expression of some cancer-inhibiting miRNA in KB cells and HepG2 cells [58]. Salicylic acid can maintain the stability of the genome and plays a key role in reducing the risk of cancer. These findings indicate that the lack of salicylic acid will lead to the delay of DNA excision and repair mechanisms, the accumulation of single-strand and double-strand breaks, cell cycle arrest, damage from apoptosis, and will increase the susceptibility to cancer development [59].

### Effects of key functional components on the viability of A549 cells

**Fig. 8:**
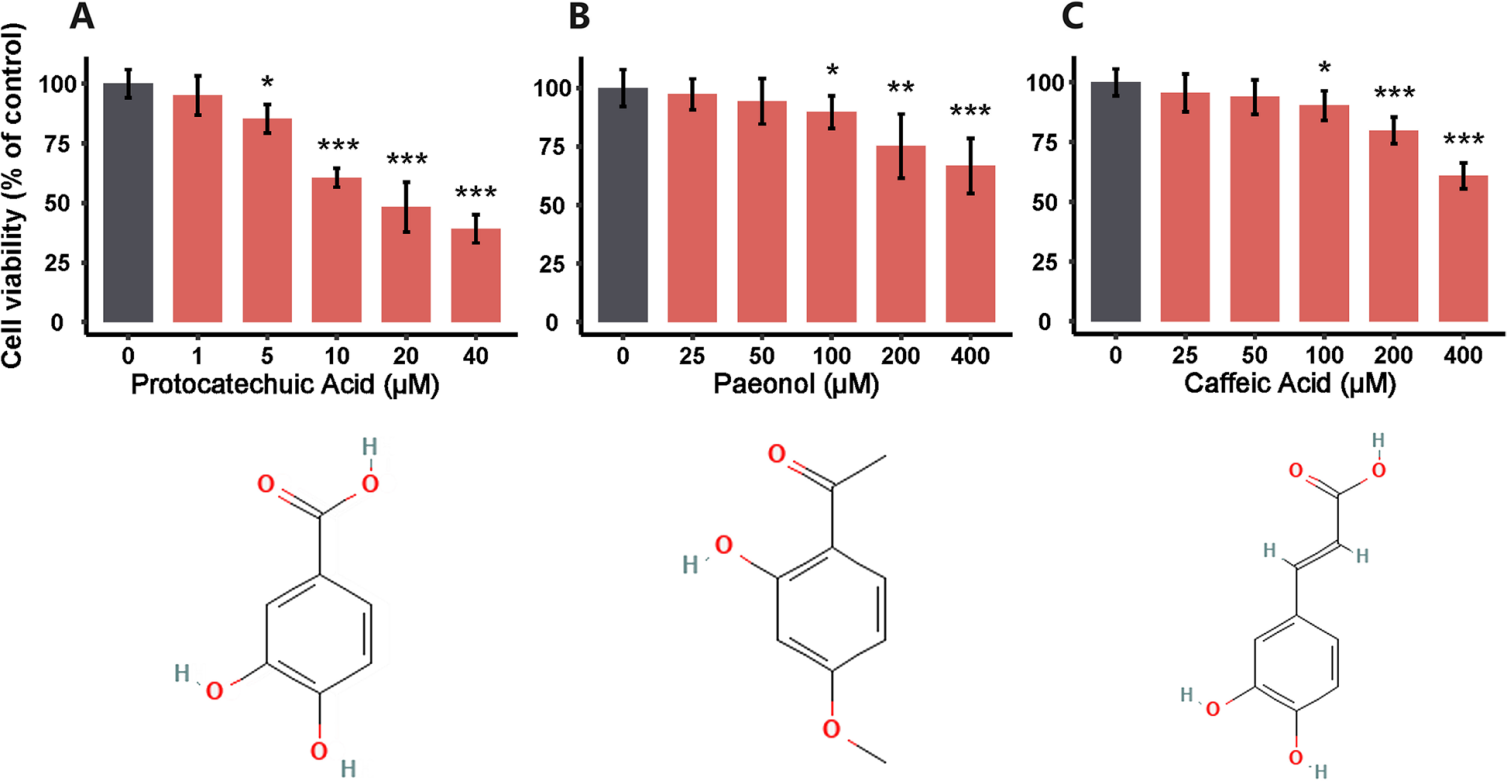
Inhibitory Effects of protocatechuic Acid **A**, paeonol **B** and caffeic Acid **C** on the proliferation of A549 cells at 24 h. Data are represented as mean ± SEM (*n* = 6). *P < 0.05, **P < 0.01, *** *P* < 0.001 versus the control group. The 2D structures are obtained from PubChem

Simple random sampling is a method of probability sampling based on chance events, and it can mitigate selection bias [60]. It’s the incorporation of randomization that provides unpredictability in treatment assignments. Based on this selective strategy, protocatechuic acid (SM-32), paeonol (SM-257), and caffeic acid (SM-229) in 82 KFC were selected to determine the effects on the viability of A549 cells using the MTT assay. Protocatechuic acid (SM-32) originates from *G. uralensis*. Paeonol (SM-257) originates from *M. alba*. Caffeic acid (SM-229) originates from *G. littoralis*. After 24 h of incubation, the cell viabilities of A549 cells were 95.07 ± 8.30%, 85.31 ± 6.08%, 60.65 ± 4.01%, 48.25 ± 10.39%, and 39.12 ± 5.88% after exposure to protocatechuic acid at concentrations of 1, 5, 10, 20, and 40 μM, respectively (Fig. 8A). After exposure to paeonol at concentrations of 25, 50, 100, 200 and 400 μM, the cell viability was 97.34 ± 6.58%, 94.33 ± 9.72%, 89.77 ± 6.94%, 75.19 ± 13.70% and 66.79 ± 11.76% (Fig. 8B). When cells were treated with 25, 50, 100, 200, and 400 μM caffeic acid, the cell viabilities were 95.60 ± 7.97%, 93.90 ± 7.26%, 90.27 ± 6.18%, 79.91 ± 5.54%, and 60.89 ± 5.39% (Fig. 8C). The results show that 5–40 μM protocatechuic acid, 100–400 μM paeonol or caffeic acid exerted significant inhibitory activity on the proliferation of A549 cells.

### Possible therapeutic mechanism

ClusterProfiler was used to perform the functional enrichment analysis of the KFC targeted genes, we obtained pathways with a *P*-value < 0.05. Among these pathways, the small lung cancer and NSCLC pathways are highly correlated with the pathogenesis of lung cancer. Increasing evidence confirms that the downstream gene regulation of the PI3K/Akt signaling pathway (hsa04151) can be changed by targeting the GPCR receptor family through PI3K and Akt. Furthermore, activation of the PI3K/Akt signaling pathway can inhibit apoptosis, promote gene transcription, and cell proliferation, accelerate the cell cycle process, and promote angiogenesis by regulating various downstream activating factors [61–63]. The MAPK signaling pathway (hsa04010) also proved to be vital to the occurrence and development of tumor, and activation of the MAPK signaling pathway may lead to increased proliferation, migration, and invasion of tumor cells [64].

**Fig. 9:**
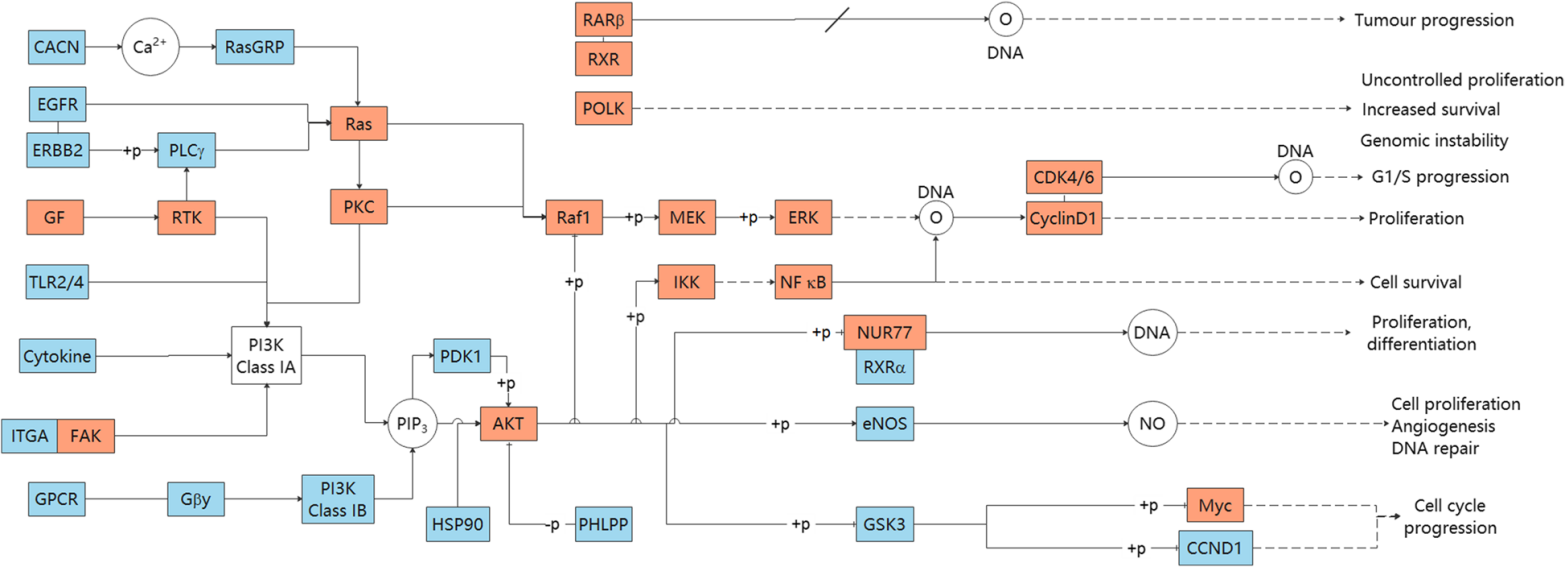
Main cascade targeting module merged by CDR-predicted cascade pathways. The red units denote the KFC targeted cross-talk genes shared by multiple pathways. The blue units indicate that the KFC targeted genes exist only in one pathway. The white units are annotations or nontargeted proteins

To further explore the synergistic effects of KFC targets in different pathways, we combined the enrichment pathways as a comprehensive pathway. The comprehensive pathway included small lung cancer (hsa05222), NSCLC (hsa05223), the PI3K/Akt signaling pathway (hsa04151), and the MAPK signaling pathway (hsa04010) (Fig. 9, Additional Fig. 2). In the combined pathways, some genes products sharing multiple pathways are named as cross-talk gene products, and main cascade targeting module merged with CDR-predicted comprehensive pathways. Some cross-talk gene products appear intermediately in merged pathways, including Ras, PKC, Raf1, MEK, ERK, CDK4/6, CyclinD1, AKT, IKK, NF-κB, and NUR77. In this module, Ras and AKT frequently regulates downstream receptors, which were also predicted as cross-talk gene products, such as Raf1, MEK, IKK, NF-κB, and so on. The downstream receptors of cross-talk gene products were reported relevant to proliferation and cell survival in other pathways. Myc and POLK, which have indirect effects on increased survival and cell cycle progression, are still worthy of attention. With the prediction of CDR model and the analysis of merged pathways, KFC could act on these targets and deprive indispensable conditions for the proliferation and long-term survival of cancer cells. In this way, therapeutic mechanism could be achieved possibly.

## Discussion

Lung cancer is the most common primary malignancy and ranks first in the incidence and mortality of malignant tumors in the world [1]. Lung cancer treatments in clinical usually present obvious side effects in clinical use, and can cause serious or general damage to specific or all tissues [2]. Many clinical studies have shown that anti-lung cancer treatment combined with TCM can reduce the side effects of radiotherapy and chemotherapy, improve therapeutic effects, and reduce complications. Meanwhile, TCM can prolong the life of lung cancer patients and improve their survival quality of life [6]. For example, SMD can alleviate symptoms of patients and can reduce the inflammatory response [10], which is widely used in the clinical treatment of lung cancer. Thus, the potential molecular mechanism of SMD in treating lung cancer was studied based on integrated network pharmacology strategy.

In this study, we designed an integrated network pharmacology strategy to explore the KFC of SMD in the treatment of lung cancer and analyze the possible mechanisms. Herbal components were obtained from TCMSP [21], ETCM [22], and SymMap [23], then potential active components were selected by ADMET properties. The targets of these active components were predicted by Hitpick [28], SwissTargetPrediction [27], and SEA [29], then the CT network was constructed. Pathogenic gene data set was downloaded from the DisGeNET. PPI data of pathogenic genes and predicted targets was obtained from STRING [30] database. Thus, a four-layer component-target-gene-disease network by combining CT, PPI network, and pathogenic gene network, was constructed. With our newly designed node-importance calculation method, key functional network was extracted. Based on knapsack algorithm, KFC were sought out to deduce the maximum target coverage of the key functional network. The accuracy and reliability of the KFC selective method was confirmed further with functional enrichment analysis and in vitro experiments.

Based on integrated network pharmacology strategy, the model designed to explore the KFC of SMD has two measurable advantages. The first is that a novel node-importance calculation model was designed to the figure out key functional networks and validate the coverage rate of key functional networks at functional level. The influence and control of a node to others have been taken into account by the node-importance calculation model, as well as the probability of a node connected to others. The results suggest that the node-importance calculation model we proposed can retain the intervention information to the greatest extent. The second advantage is that we develop the CDR model to capture the KFC in key functional networks. Compared with the traditional pharmacology method based on one-way target speculation mechanisms, the main innovation of our model is that it can provide a methodological reference for the development of integrated pharmacology by considering the spread of the intervention effect from targets to pathogenic genes.

With the prediction of CDR model and the analysis of merged pathways, cross-talk gene products were found, and main cascade targeting module merged with CDR-predicted signalling pathways was visualized clearly. Some cross-talk gene products appear intermediately in merged pathway. Among them, Ras and AKT frequently regulates downstream receptors and other cross-talk gene products, affecting proliferation and cell survival in other pathways. Other cross-talk gene products are still worthy of attention, such as Myc and POLK, despite indirect effects on increased survival and cell cycle progression as reported. After computational analysis and in vitro experiments, KFC could act on these cross-talk gene products to treat lung cancer, and possible therapeutic mechanism could be decoded.

Compared with the traditional pharmacology method based on one-way target speculation mechanisms, the main innovation of our model is that it can provide a methodological reference for the development of integrated pharmacology by considering the spread of the intervention effect from targets to pathogenic genes.

However, it still exists two limitations in our study. First, concentrations of herbal components, that could measure the effects of drug intervention network more exactly, are ignored in our network pharmacological analysis. Second, in order to validate the reliability of our approach, more KFC should be selected to determine the effect of viability of A549 cells or in vivo experiments.

## Conclusions

In conclusion, the molecular mechanism of SMD treatment of lung cancer was revealed by integrated network pharmacology model and experimental validation. The strategy proposed in this study can be used to identify key compounds in the complex network and provides an operable test range for subsequent experimental verification, which greatly reduces the experimental workload. In follow-up studies, in vitro and in vivo animal studies can be designed based on the predicted KFC.

## Supplementary Information

**Additional file 1.** Supporting Information.

**Additional file 2: Additional Table 1.** All herbal components of SMD. **Additional Table 2.** Active components of SMD. **Additional Table 3.** Predicted targets of active components from SMD. **Additional Table 4.** Pathogenic genes of lung cancer downloaded from DisGeNET database. **Additional Table 5.** The CDR of active components.

**Additional file 3: Additional Figure 1.** Construct complex components-targets-pathogenic genes-disease (C-T-P-D) network. Green units represent active components of SMD, blue units represent predicted targets of active components and red units represent predicted pathogenic genes of lung cancer. The gray lines indicate the interactions.** Additional Figure 2.** Full distribution of KFC targets on merged paths. The red units denote the KFC targeted cross-talk genes shared by multiple pathways, and the red dashed line connects them together in different pathways. The blue units indicate that the KFC targeted genes exist only in one pathway. The white units are annotations or nontargeted proteins.

## Data Availability

All data included in this study are available upon request by contact with the corresponding author. Human data in this study was obtained from public database DisGeNET (https://www.disgenet.org/).

## Abbreviations

ADMET: Absorption, Distribution, Metabolism, Excretion, and Toxicity
CDR: Contribution Decision Rate
CI: Contribution Index
CTP: Components-Targets-Pathogenetic genes
DL: Drug-Likeness
DMEM: Dulbecco’s Modified Eagle’s Medium
FBS: Fetal Bovine Serum
GO: Gene Ontology
*G. littoralis*: *Glehnia littoralis* (A.Gray) F.Schmidt ex Miq.
*G. uralensis*: *Glycyrrhiza uralensis* Fisch. ex DC.
ICD: International Classification of Diseases
KFC: Key Functional Components
KEGG: Kyoto Encyclopedia of Genes and Genomes
*L. purpureus*: *Lablab purpureus* Subsp. *purpureus*
*M. alba*: *Morus alba* L.
NSCLC: Non-Small Cell Lung Cancer
*O. japonicus*: *Ophiopogon japonicus* (Thunb.) Ker Gawl.
OB: Oral Bioavailability
*P. odoratum*: *Polygonatum odoratum* (Mill.) Druce
PPI: Protein–Protein Interactions
SMD: Shashen Maidong Decoction
SEA: Similarity Ensemble Approach
SymMap: Symptom Mapping
TCM: Traditional Chinese Medicine
TCMSP: Traditional Chinese Medicine Systems Pharmacology Database and Analysis Platform
*T. kirilowii*: *Trichosanthes kirilowii* Maxim
