# Supplementary material for: Decoding the key compounds and mechanism of Shashen Maidong decoction in the treatment of lung cancer
Source: BMC Complement Med Ther. 2023 May 15;23:158. doi: 10.1186/s12906-023-03985-y (PMC10184424; doi:10.1186/s12906-023-03985-y)
Supplement: Supplementary file 1 — Additional file 1. Supporting Information. [file 12906_2023_3985_MOESM1_ESM.docx]

## Supporting Information

### The algorithm of screening key functional networks

The algorithm is described as follows:

$$\emptyset=\max_{i<n} (d_{1\to2},d_{1\to3},d_{1\to4}\cdots d_{i\to j}\cdots d_{(\frac{n\left( n-1 \right)}{2}-1)\to\frac{n\left( n-1 \right)}{2}})$$

$${IM}_{i}=\sqrt{\frac{\left( \emptyset+1 \right)-{\sum d_{jk}(i)}/m}{\emptyset}\times\frac{\sum_{j}^{n} \sum_{k}^{n} \frac{g_{jk}(i)}{g_{jk}}}{n(n-1)/2}}$$

$$IM_{median}=median\left\{ {IM}_{1}, {IM}_{2},{IM}_{3},\cdots,{IM}_{n} \right\}$$

$$\text{IntS=}\bigcup_{i=1}^{n} {IM}_{\left( Net_{ctpd} \right)i}>{IM}_{median}$$

IMi indicates the importance of node i in the network; ∅ is the maximum distance between two nodes in the network. And ∅ is the maximum distance between all connected components in the disconnected network. g_jk_ indicates the number of paths between nodes j and k. g_jk_(i) is the number of paths from node j to node k and through node i. d_jk_(i) is the number of shortest paths passing through node i from node j to node k; m is the total number of shortest paths passing through node i in the whole network; n represents the total number of nodes in the network. IntS stands for the key functional network.

### The algorithm of predicting key functional components

The algorithm is as follows:

$$\begin{matrix} \text{For }x\in(1:n)\text{ do} \\ \text{if }C_{kx}=\max(C_{k1}:C_{kn})\text{ then} \\ \prod_{k1:k(n-1)} =\prod_{k1:kn} -k_{x} \\ \prod_{k(x+1)} =\prod_{kx} +k_{x} \\ \text{else if }C_{kx}=C_{ky}=\max(C_{k1}:C_{kn})\text{ and }U_{kx}\geq U_{ky}\text{ then} \\ \prod_{k1:kn} =\prod_{k1:kn} -\prod_{kx+ky} \\ \prod_{kx} =\prod_{kx} +k_{x} \\ \text{End if} \\ \text{C}_{\text{U}_{\text{kx}}}\geq90\% \\ \text{End if} \\ \prod_{k1:kn} =\emptyset\\ \text{Return} \\ \text{KFC}=\prod_{kx} \end{matrix}$$

k represents the component in SMD; kn represents the nth component; U represents unite; U_k_ represents the unit of components; U_kn_ represents the target unit of the nth components; C represents the coverage of targets in U_j_.
