# Supplementary material for: Decoding the key compounds and mechanism of Shashen Maidong decoction in the treatment of lung cancer
Source: BMC Complement Med Ther. 2023 May 15;23:158. doi: 10.1186/s12906-023-03985-y (PMC10184424; doi:10.1186/s12906-023-03985-y)
Supplement: Supplementary file 3 — Additional file 3: Additional Figure 1. Construct complex components-targets-pathogenic genes-disease (C-T-P-D) network. Green units represent active components of SMD, blue units represent predicted targets of active components and red units represent predicted pathogenic genes of lung cancer. The gray lines indicate the interactions. Additional Figure 2. Full distribution of KFC targets on merged paths. The red units denote the KFC targeted cross-talk genes shared by multiple pathways, and the red dashed line connects them together in different pathways. The blue units indicate that the KFC targeted genes exist only in one pathway. The white units are annotations or nontargeted proteins. [file 12906_2023_3985_MOESM3_ESM.docx]

Additional Figures


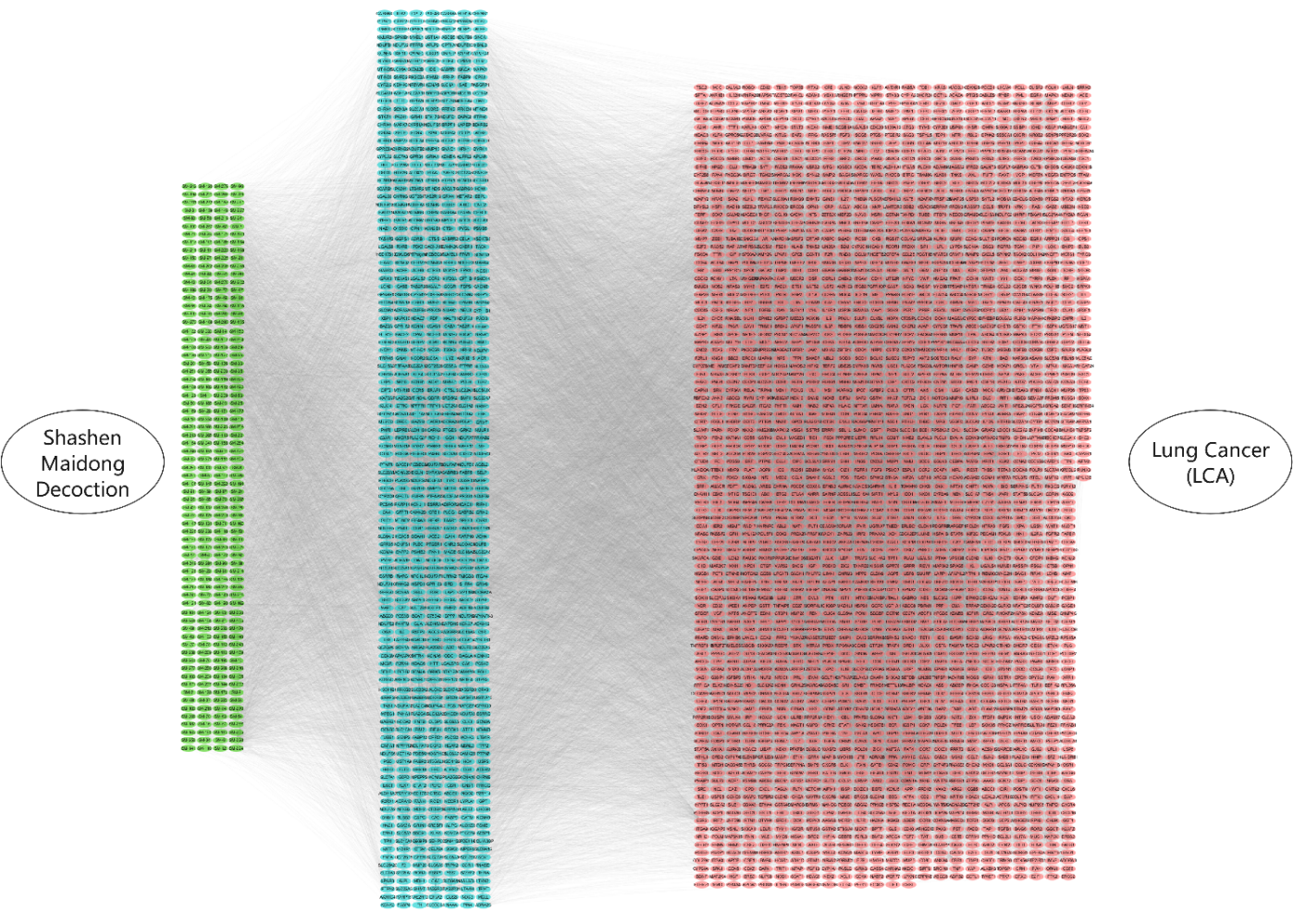


**Additional Figure 1.** Construct complex components-targets-pathogenic genes-disease (C-T-P-D) network. Green units represent active components of SMD, blue units represent predicted targets of active components and red units represent predicted pathogenic genes of lung cancer. The gray lines indicate the interactions.


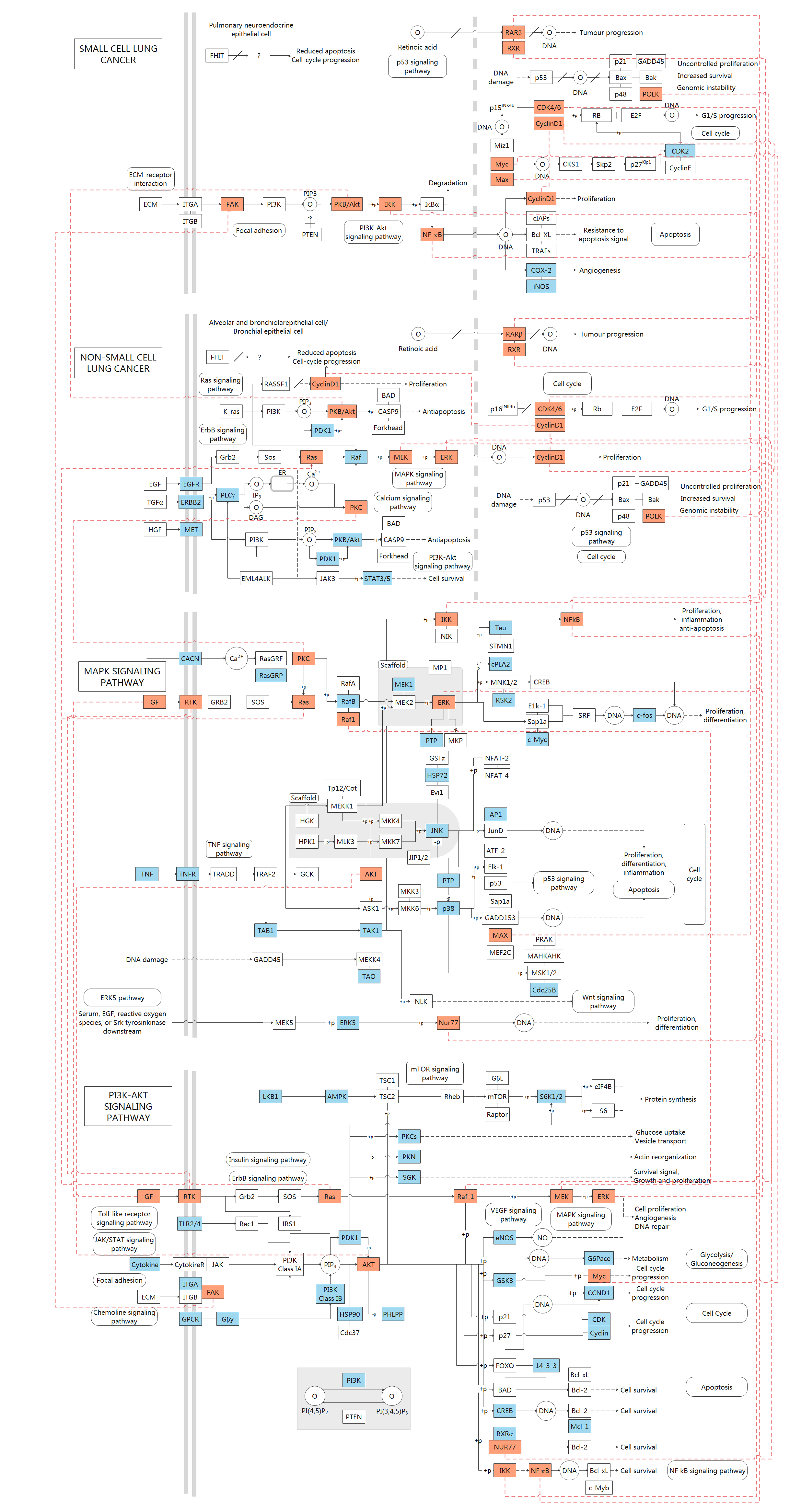


**Additional Figure 2.** Full distribution of KFC targets on merged paths. The red units denote the KFC targeted cross-talk genes shared by multiple pathways, and the red dashed line connects them together in different pathways. The blue units indicate that the KFC targeted genes exist only in one pathway. The white units are annotations or nontargeted proteins.
